# User-Centered Design Process of an mHealth App for Health Professionals: Case Study

**DOI:** 10.2196/18079

**Published:** 2021-03-26

**Authors:** Amarasinghe Arachchige Don Nalin Samandika Saparamadu, Piyum Fernando, Peizi Zeng, Henry Teo, Andrew Goh, Joanne Mee Yin Lee, Choong Weng Leslie Lam

**Affiliations:** 1 MOH Holdings Private Limited Singapore Singapore; 2 School of Arts, Media, and Engineering Arizona State University Tempe, AZ United States; 3 Department of Laboratory Medicine Ng Teng Fong General Hospital Singapore Singapore

**Keywords:** user-centered design, participatory design, mobile health applications, mHealth, smartphones, health professionals, healthcare, human-computer interaction, mobile phones

## Abstract

**Background:**

User-centered design processes are infrequently employed and not fully explored for building mobile health (mHealth) apps that are particularly targeted to health professionals as end users. The authors have used a user-centered design–based approach to build an mHealth app for health professionals, tasked to deliver medical laboratory-related information on a daily basis.

**Objective:**

Our objective is to generate a simple and functional user-centered design process for mHealth apps for health professionals. This paper presents the key learnings from design activities.

**Methods:**

A stratified random sample of doctors and nurses was recruited for the study. The design activities were planned in the following sequence: focus group discussion for situation analysis and information architecture, design activity 1 for wireframe designing, design activity 2 for wireframe testing, and user testing sessions 1 and 2.

**Results:**

The final design and functions of the app, information architecture, and interactive elements were largely influenced by the participatory design–based user-centered design activities. As a result of the design process, we could identify the mental models of processing requests for information and personal preferences based on the experience. These findings were directly or indirectly incorporated into the app design. Furthermore, finding alternative ways of working within time constraints and cultural barriers and the methods employed to manage the challenges of interdisciplinary discourse stood out among the lessons learned.

**Conclusions:**

We recommend a user-centered design process based on a participatory design approach in mHealth app design, enriched with focus group discussions where possible.

## Introduction

### Background

The term mobile health (mHealth) is defined as emerging mobile communications and network technologies for health care systems, which is an exponentially growing market in the smartphone era [1,2]. Current estimates suggest that there are more than 40,000 mHealth apps [3]. The global mHealth market is projected to grow at a compound annual growth rate of around 35.65% over the next few years to reach approximately US $115.61 billion by 2025 [4].

Many current mHealth interventions for health care–related issues are designed on the basis of existing healthcare system constructs, and they may not be as effective as those that involve end users in the design process [5]. In recent times, user-centered design–based approaches have been reportedly used for building mHealth apps, most of which are focused on chronic diseases [6-9], cancer [10,11], health and well-being, lifestyle interventions [12-14], mental health [15], sexual health [16], pain management [10,17], and remote patient monitoring [18]. Commonly used user-centered design frameworks for building mHealth apps include Information Systems Research [19], the Health Information Technology Usability Evaluation Model [20], and the BUS (behavior change theories, user-centered design, and social marketing) framework [21].

Despite the growth of user-centered design–based mHealth apps built for patients and the general public, there has been little exploration of how to use them for health professionals as end users. Hence, little is known about the unique challenges and opportunities for applying user-centered design to build apps for health professionals.

Our findings are drawn upon a real-world design process where the authors have employed a participatory design–based user-centered design approach to build an mHealth app targeting health professionals. Traditionally, laboratory-related information is made available to the staff through a laboratory service manual, which is a 217-page document accessed via the hospital intranet, describing over 525 laboratory investigations and procedures available at the hospital. The idea of delivering this information through an mHealth app was conceived as a result of an audit conducted to describe the phone call patterns received at the laboratory and a follow-up survey conducted among health professionals that revealed the towering demand for laboratory-related information and the infrequent use of the laboratory service manual by end users who were inclined toward an mHealth intervention [22]. Combined, these findings set out the primary objective of this app, which is to replace the existing laboratory service manual and to create an efficient, convenient platform to deliver medical laboratory-related information to health professionals at Ng Teng Fong General Hospital, Singapore, on a daily basis.

Health professionals’ frequent dilemmas related to laboratory tests in clinical practice take many forms: deciding the adequacy of the size of a specimen according to laboratory’s rejection criteria, clarifying the correct method of collection and specimen container, decisions related to the urgency of investigations and results, etc. In these scenarios, laboratory-related information such as specimen type, minimum sample size, appropriate specimen container or tube, method of collection, special instructions (eg, fasting blood sample), turnaround time, price, etc, play an important role in decision making.

The contribution of this paper is twofold: first, we describe our methods, materials, results, and outcomes. Then, by reflecting on our own experiences, we discuss the insights and implications of our work through the challenges faced, lessons learned, and strategies adopted to overcome challenges.

### Objectives

Our objective is to generate a simple and functional user-centered design process for mHealth apps for health professionals (specifically, for an app that delivers medical laboratory-related information).

## Methods

### Overview

To achieve our objective, we aimed to employ (a) an intermediate approach to user-centered design and participatory design by direct and indirect involvement of our end users (doctors and nurses) at various stages of the design process and (b) multiple user-centered design methods and to tailor the whole process according to the unique needs of our end users. In this section, we describe the methods in sampling, data analysis, and designing activities and sessions, as well as the design procedures.

**Figure 1:**
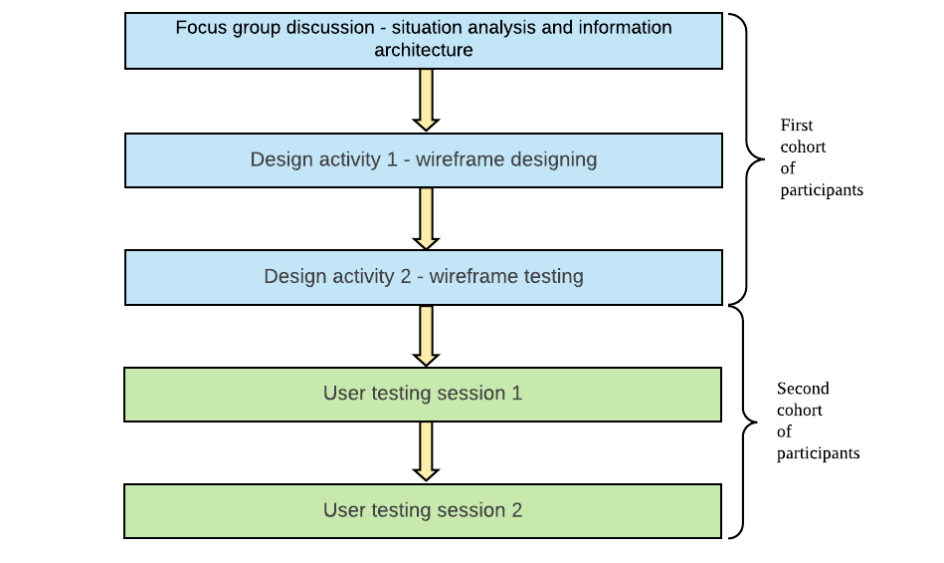
An illustration of the sequence of design activities and sessions.

A stratified random sampling model was employed by the study team—the project team and a human-computer interaction (HCI) consultant—to select participants for the sessions. Strata were defined to include inpatient settings (medical, surgical wards, and the ambulatory unit) and outpatient settings or clinics. We recruited doctors and nurses in rough proportion to their workforce ratios. However, the emergency department was excluded in view of significant time constraints and perceived manpower shortage (Table 1). Informed written consent was obtained from all participants throughout the process prior to each session or design activity (Figure 1).

**Table 1:** Demographic data of participants.

Characteristic		First cohort (focus group discussion and design activities)	Second cohort (user testing sessions)	Total
Participants, n		15	9	24
Age (years), mean (SD)		35.4 (8.1)	34.8 (7.6)	—^a^
**Gender, n (%)**				
	Female	12 (80)	6 (67)	18 (75)
	Male	3 (20)	3 (33)	6 (25)
**Nationality, n (%)**				
	Singaporeans	9 (60)	2 (22)	11 (46)
	Non-Singaporeans	6 (40)	7 (78)	13 (54)
**Ethnicity, n (%)**				
	Chinese	7 (47)	3 (33)	10 (42)
	Malay	3 (20)	1 (11)	4 (17)
	Indian	3 (20)	1 (11)	4 (17)
	Others	2 (13)	4 (44)	6 (25)
**Role in hospital, n (%)**				
	Physician	5 (33)	2 (22)	7 (29)
	Nurse	10 (67)	7 (78)	17 (71)
**Setting, n (%)**				
	Inpatient/ward	9 (60)	6 (67)	15 (62)
	Outpatient clinic	4 (27)	0 (0)	4 (17)
	Ambulatory clinic	2 (13)	3 (33)	5 (21)

^a^Not available.

Statistical analysis of demographic data was carried out on IBM SPSS, version 23.0 (IBM Corp). Categorical variables were expressed as frequencies and percentages, and continuous variables were expressed using means and standard deviation.

Following each session, all study data, including audio recordings and handwritten notes, were transcribed verbatim without participant identifiers and annotated with pauses and nonverbal expressions. Anonymized transcripts were analyzed by the study team to create and examine codes, themes, and categories. Transcripts were coded independently by two individuals using line-by-line coding technique. The codes were compared, and the differences were discussed among them. Affinity mapping technique was employed for thematic analysis and establishing categories, and the findings, outcomes, and insights were generated based on this analysis.

To further ensure reliability of the study, effective session facilitating techniques were utilized: well-prepared facilitators (social and conversational skills) and rapport building with the participants.

### Focus Group Discussion: Situation Analysis and Information Architecture

The objective of the first session was to understand the existing user behavior and practices related to laboratory information usage (situation analysis) and obtain initial user inputs to determine what laboratory information should be presented and how it should be structured and highlighted in the app (information architecture). In addition, we used this session as a means of building rapport and goodwill between participants and the facilitators.

This session was designed to be conducted in a span of an hour. First, we randomly divided all the participants into three subgroups by counting numbers; the same method has been used in the activities mentioned throughout the paper. In the first activity, the participants were given a scenario of a less frequently used laboratory investigation requested by a physician during a busy morning ward round. We asked them to write down the first things that came to their minds on sticky notes while they were fulfilling this task. We requested them to produce at least five responses per group. Following this activity, one group member from each group was asked to share the responses on the whiteboard.

In the next activity, the same groups were asked to draw a flowchart to explain the process of dispatching a blood sample as part of their routine daily work. Groups were given an example of how to perform this task, and they were encouraged to simulate a real-life scenario of their choice. Members were asked to describe their flowcharts to other groups and were given the opportunity to ask questions.

The third activity was designed to prioritize the information available in the laboratory service manual to be included in the app based on user input. We provided details of 10 uncommon laboratory tests available in the laboratory service manual and asked participants to use color codes (red, yellow, and green) to highlight information based on following criteria: green for details that they already knew from memory, red for details that they did not know unless they referred to the manual, and yellow for details for which they needed reassurance or confirmation. We carefully handpicked 10 tests, considering the scarcity of information on the type of specimen, tube or container, use of preservatives or transport media, dispatch instructions, other special instructions, etc.

Finally, we prioritized the information based on the user responses using a four-quadrant matrix under two main domains (how frequently it is used and how well it is known to the user) in order to summarize them (ie, mostly known vs least known and least used vs mostly used).

### Design Activity 1: Wireframe Designing

The first design activity was planned in the form of a co-design activity. The same cohort of participants was present in the second session, except for 1 participant who had called in sick. She was replaced by another member from the same ward.

This session was an hour-long design activity. It consisted of three main activities focused on determining the information architecture and designing the initial wireframes (skeletal visual representations of the app screens). Participants were randomly divided into two groups, and each group was given a set of selected laboratory investigations written on A5-size papers. It had a balanced representation of the mainstream disciplines of laboratory medicine: chemical pathology, microbiology, anatomic pathology, hematology, immunology, genetics, and molecular diagnostics. The set of tests given to each group were not identical but comparable. They were asked to classify all the tests handed to them within 10 minutes. Following this, they were asked to share the principles of the classification that they employed, and each group was given a chance to ask questions of the other group. Both groups were given another chance to reclassify investigations in a different way and in a shorter period of time (5 minutes).

Following this activity, we asked participants to jot down at least five different keywords that they would use to search for each investigation on the reverse side of each paper. This was aimed at gathering user inputs toward designing the search function of the app.

In the third activity, both groups were asked to sketch wireframes depicting how they envisioned the app's home page, visual representation of test details, and search functionality. An in-depth discussion was carried out to understand the reasons behind each wireframe design.

### Design Activity 2: Wireframe Testing

The same cohort of participants from the previous design activity attended the third session, with the addition of 2 new participants. Data from the previous session were analyzed and discussed with the study team. Based on the outcomes of the previous session, two sets of wireframe mockups were created by the HCI consultant: one with a home page containing the categories of tests (categorized by discipline and by specimen type) and a directly accessible list of frequently used tests and the other with a home page with links to go to “categories” and “all tests” and a list of frequently used tests.

Once again, the participants were randomly divided into two groups. An open discussion on design, ease of use, order and presentation of information, user-friendliness, and specific functions was conducted among groups with open-ended questions (Table 2). We also asked some specific questions that the project team and HCI specialist had during mockup wireframe preparations to shed some light on uncertainties.

**Table 2:** Questionnaire for design activity 2, wireframe testing.

Serial number			Script
**Home page**			
	1	Please go to the home page [hand them the first prototype] and tell me how you would feel if you got to use this design/user interface on a smartphone app?	
	2	What do you think about this home page design?	
	3	Think of/give some example tests that you would like to add to this list. How often do you think you would access a test using this list? Where do you like to have the list of bookmarked tests in your mobile app? Why do/don’t you think it is useful to have this list on the home page?	
	4	Will an “all tests” function be useful? Alternatively, would you prefer to access “all tests” using the search icon below? Why?	
	5	How do you want to see new notifications on your app? How important are these notifications to you?	
	6	[Now, the interviewer hands them the second prototype of the home page.] What do you think about this design/user interface?	
	7	What do you think about this design compared to the previous one?	
	8	Do you think that you need an icon for “most common tests” that would by default have some commonly used tests across all disciplines listed in it and would be customizable [you can add or remove items from this list]? Alternatively, do you want something like “my tests,” which would come empty with the app but be customizable according to your needs?	
**Categories**			
	1	These categories were carefully selected depending on your suggestions and discussions from the last week. What are your thoughts about this categorization? [Repeat this question for each categorization.]	
	2	Do you have any other suggestions for categorizations?	
	3	Can you take a few minutes and think of any test that would not easily fit into any of these categories? [Please explain that the idea of the categories is fluid and one test could appear in more than one category if necessary, overlapping.]	
	4	Any other comments/suggestions?	
**Search function**			
	1	As per our discussions from the last session, we realized that the search function should be robust. Here are the shortlisted “keywords” in order of importance that are suggested to be used in the smart search engine: medical domain/discipline and related diseases. [Interviewer gives a real-life scenario.] What do you think about this search order? What are the changes/modifications you would like to introduce to this search function?	
	2	Do you want to see your old search items and history before you start your search? Why?	
	3	Any other comments/suggestions?	
**Test-details page**			
	1	What do you think about the arrangement of information under each test [ask about spacing, font size, graphics, pictures of the tube, etc]?	
	2	Any comments on the order of information? Any suggestions to change this interface?	
	3	Is there anything else that you would like to see on this page [information or graphical presentation]?	
	4	What do you think about the picture of the tube? Any other details that you would like to see in this image?	
	5	How do you want to add this test into your bookmarked list? What is your preferred workflow?	
	6	This design uses a “tick” on the right upper corner. What kind of icon (or user interface element) would you prefer?	
**Hamburger menu**			
	1	[A wireframe of a hamburger menu is to be handed to each group.] Could you try and list the things that you want to see/ would like to see on this page?	
	2	[Once participants have finished listing, facilitate a discussion on each listed item.] Why do you want this item on the hamburger menu, and what are the reasons why it’s best suited in the menu page but not elsewhere (ie, home page)?	

### User Testing Session 1

Within 5 months from the third session, the first functional prototype of the app was developed based on the user inputs gathered since inception. The first user testing session was conducted to evaluate the usability of the first functional prototype. An all-new group of nurses and doctors were recruited using stratified random sampling methods. Out of 10 participants, 5 used iOS and 5 used Android operating systems. One Android user dropped out of the study due to privacy concerns related to installing an unpublished external app on the phone. This incident did not have an impact on the final design of the app as the process of publishing spontaneously resolves the concern.

All the participants were given a copy of the prototype laboratory mobile app downloaded on their mobile phones. They were asked to perform a set of specific tasks in the shortest possible time, and the time taken by each participant was recorded. A discussion was carried out with each group using open-ended questions. Furthermore, at the end of the session, we obtained feedback from the participants on general topics (Table 3).

**Table 3:** Questionnaire for user testing session 1.

Serial number			Script		Allocated time (min)
**Verbal and visual instructions given to all participants**					13-15
	1	Find out “special instructions” for taking a blood culture.			
	2	Assume that you find a piece of false information on a “test page.” Submit feedback regarding this issue using the app. [E.g., a test page of full blood count says that the sample has to be sent in a “yellow tube.”]			
	3	Find the contact information to arrange a “frozen section.”			
	4	Search and find a test to help diagnose Wilson disease. [Hint: You can probably find this test on the app even if you do not remember the name of the test.]			
	5	Go to categories “by discipline,” create a new category, and give that new category a name. Then, add three (3) tests of your choice into the new category that you created.			
	6	Go to the category that you created and remove one (1) test from the category.			
	7	Find the “order of draw” on the app.			
	8	Go and find the types of tubes/containers that you can use to send specimens to check “acetaminophen level” of a patient.			
	9	Check how many phone notifications you have received on your app.			
**Questions to be asked of participants after each task is performed**					25
	1	Tell us about your experience of performing this task.			
	2	What are the difficulties you faced while performing this task?			
	3	I like [the things that I like about this feature/function].			
	4	I wish [the things that I wish to see in this feature/function].			
	5	Ask more questions in detail depending on the scenario (eg, What are your concerns regarding the current design? How would you suggest improving it? What would you like to see instead of the current design/workflow?).			
**General thoughts to be asked at the end of the session**					5-10
	1	Any further suggestions to improve the functionality of the app?			
	2	What do you think about the information provided on the app? Do you see anything missing? Do you find anything that is not useful?			
	3	How do you like the look and feel of the app, such as the colors, fonts, and user interface elements like buttons and menus? Any suggestions to improve them?			
	4	Are there any other thoughts/suggestions/comments you would like to add that have not been discussed so far?			

### User Testing Session 2

Within 3 months from the first user testing session, the second prototype of the app was prepared. The same cohort of participants who took part in user testing session 1 were invited to take part in this activity. A total of 9 participants took part in this session.

Participants were asked to download a copy of the latest version of the laboratory mobile app, to use it at work, and to try to replace their daily work process with the app. After allowing them to use the app for a week, we interviewed them individually using an open-ended questionnaire.

## Results

### Overview

The mean ages of our participants were 35.4 and 34.8 years for the group that participated in focus group discussions and design activities and for the group that participated in user testing sessions, respectively. The youngest member of the whole cohort was a 24-year-old medical officer, while the oldest member was a 53-year-old assistant nurse clinician. Singapore being a multiethnic, multicultural city-state, we observed a representation of all three major ethnic groups (ie, Chinese, Malay, Indian) as well as a fair representation of migrant workers who contribute to Singapore health care from the following countries of origin: China, India, the Philippines, Malaysia, South Korea, and Sri Lanka (Table 1). Moreover, all members of the groups were smartphone users, and all of them had more than one year of experience with the electronic medical records of the hospital, Epic System (Epic Systems Corporation).

### Focus Group Discussion: Situation Analysis and Information Architecture

From this session, we identified existing behaviors and habits of our users. We also observed significant differences in the use of terminology and work processes between nurses and doctors, which in turn generated valuable insights into ensuring a greater level of customizability of the app. In addition, we identified that the levels of importance related to variables of laboratory tests (ie, test details) are understood by all subgroups in similar patterns, making certain pieces of information salient while making the remainder less important. This information was directly used in determining the visual layouts of the ‘test-details’ page.

### Design Activity 1: Wireframe Designing

**Figure 2:**
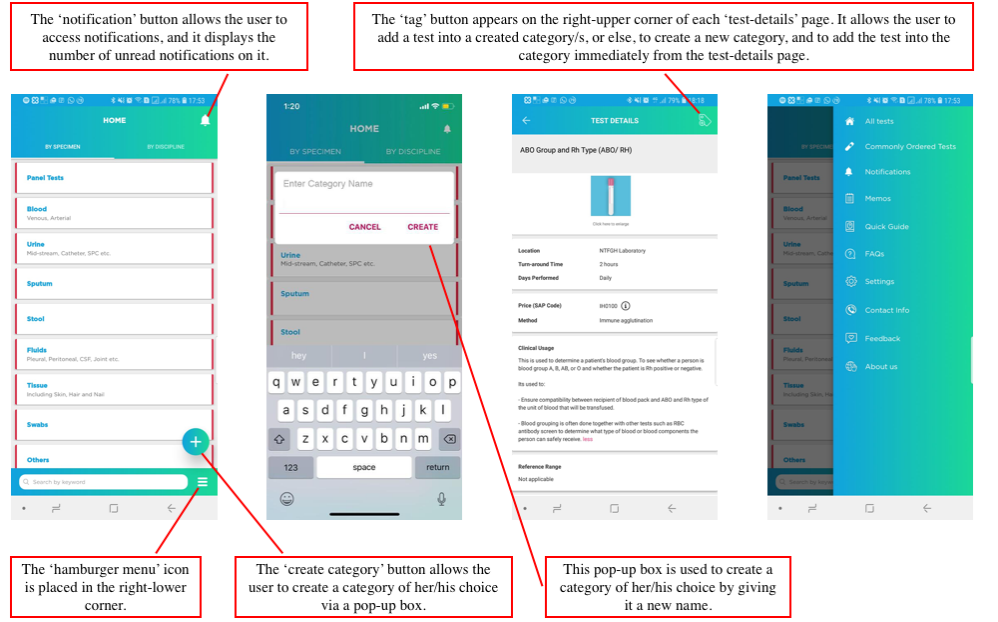
First version of the laboratory mobile app after focus group discussions and design sessions.

These activities helped to foreground two commonly used criteria to classify laboratory tests: by specimen and by discipline. These outcomes were directly incorporated into the design of the home page (Figure 2). Furthermore, nurses preferred to search for investigations by the specimen whereas doctors preferred to search by the related discipline; however, both groups preferred to have their own lists of investigations to refer to instantly, as well as a smart search engine.

Surprisingly, the wireframe designs of the “home page” and “test-details” page were strikingly similar between the two groups. They both suggested to include categories of tests and a search button on the home page, assuming that users will mostly use the search button instead of looking for tests in categories, and a starred or quick access list. Both groups prioritized details such as special instructions, pictures of relevant test tubes, containers, and preservatives, and the price of the tests, in that order. One group suggested that it would be useful to display a list of related tests under each test, and the other group was in agreement.

Participatory wireframe design activities helped the study team to further identify the features that can be useful for users. For example, a periodically updated list of commonly ordered tests was eventually added to the app based on the ideas generated from wireframe designing sessions, as it can be useful for new staff as reference material.

### Design Activity 2: Wireframe Testing

Wireframe testing triggered the participants to express their preferences and comment on potential improvements that can be made to the wireframe designs presented to them. For example, participants preferred to see a home page with all the test categories displayed on it, as opposed to the alternative wireframe. In addition, reference ranges were added, and a photograph of the tube or container was suggested over an illustration with regard to the “test-details” page. Outcomes of the third session are shown in Figure 2.

### User Testing Session 1

**Figure 3:**
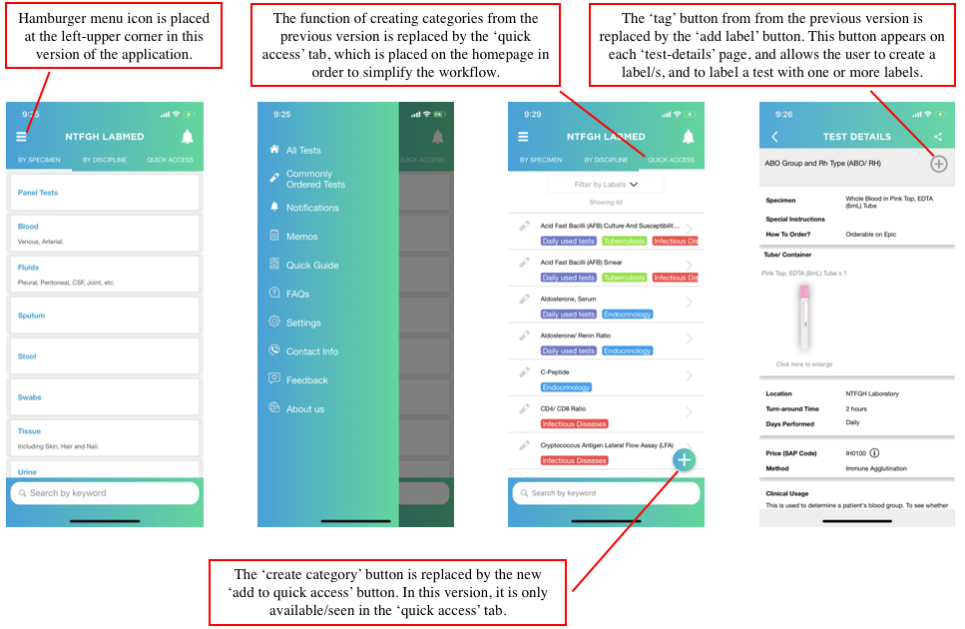
Second version of the laboratory mobile app after user testing sessions.

We identified the specific pain points for users when they perform certain tasks with the mobile app through this session. For example, users pointed out the process of creating categories as a cumbersome workflow and suggested changes. In addition, they suggested moving the hamburger menu icon that was placed on the right-lower corner to the left-upper corner, as it is commonly observed in popular local e-commerce apps. Outcomes of the fourth session are shown in Figure 3.

### User Testing Session 2

The second user testing session revealed that the users were generally satisfied with the functions and user interface of the app, and there were no specific suggestions to ameliorate the design (Figure 3). Hence, it was decided by the project team that the app was ready for launch.

## Discussion

### General Takeaways

The final design of the app, including the features, functions, information architecture, interactive elements, and aesthetics, were largely influenced by the participatory design–based user-centered design activities. As a result of the design process, we could identify the existing mental models of the end users related to laboratory tests, level of significance of laboratory-related information, how requests for information are dealt with, and how additional information is requested via telephone calls. There were personal preferences depending on the knowledge base, experience, setting, and job scope. These findings were directly or indirectly incorporated in the final design of the app. Furthermore, it triggered the need for in-depth customization while maintaining generally used mental models in the user interface of the app.

In parallel to the outcomes of the user-centered design activities, the final design of the app was influenced by the inputs from the HCI specialist based on universal user experience design principles. These inputs were incorporated in implementing several key features of the app. For example, simplified illustrations of specimen containers (eg, test tubes) were used in place of actual photographs to avoid information overload and to emphasize distinct features of each container type. Similarly, a quick access one-touch search function and the ability to assign custom labels to tests were also added based on the HCI specialist’s suggestions.

By reflecting on our user-centered design process and our own experience of working with multiple stakeholders including the project team, an HCI consultant, health professionals, and an external developer team, we discuss key takeaways of this case study under four categories below.

### Cultural Considerations

Despite the hierarchy that is observed in the medical culture [23], we observed more collaborative work and interdependency during design activities and discussions. However, it was observed that mostly doctors started leading the group during the technical discussions including information architecture, and nurses were eager to get the doctors involved. During wireframe testing and participatory design activities, the nurses were more inclined to pen down their ideas, and doctors were more collaborative despite leading the group. Disagreements between group members were dealt with through ongoing discussions; however, in certain instances we observed outlying ideas being rejected by doctors and experienced nurses without their merits being carefully examined.

These behavioral patterns were prominent during the first session, but during design sessions, they were noted to have eventually diminished. Possible reasons could be enhanced group dynamics over the course of the sessions and the nature of activities during different sessions. For example, the first focus group discussion was more dependent on technical knowledge and experience, whereas medical or technical knowledge did not have a bearing on the design activities.

### Designing the Study Around Constraints

While attempting to make focus group discussions, design sessions, and user testing sessions equally accessible and ensure fair participation, we faced major challenges with time constraints, making it the most significant challenge to mention [24,25].

In response to these legitimate limitations, the study team conducted a mock focus group discussion with elective nursing students who were rotated to the Department of Laboratory Medicine as part of their polytechnic training. Furthermore, facilitators trained themselves with each other while giving running commentary and feedback. These activities were carried out to see how well the participants could understand quick instructions, as well as to evaluate timeliness of activities and discussions. Mock focus group discussions helped us to fine-tune the construction and wording of questions and instructions to better relate with our participants culturally [26,27] and to make them succinct to save time.

We also carried out a quick feedback activity after each focus group discussion and design session to better understand the concerns of the participants. As a result of the feedback, snacks were arranged for subsequent sessions, which further encouraged participation. It was also conveyed by the participants that it’s important to commence and conclude sessions right on time in view of serious time constraints that they were facing [24,25].

### Benefits of Participatory Design Approach for Designing Information Architecture

A well-designed information architecture helps users find information efficiently and complete tasks easily (ie, finding information on a particular blood test by navigating through the app user interface). In our case, information that is intended to be presented in the proposed mHealth app (ie, laboratory test information) has already been stored in end users’ memories to various degrees as they refer to that information during daily hospital routines. Therefore, the key goal of co-designing the information architecture with end users was to understand their existing mental models of this information and try to build upon them. The outcomes of the co-design activity, where the end users categorized and labeled a selected set of laboratory tests, provided valuable insights to this end.

While there are many instances where co-designing approaches have been successfully utilized for designing information architecture [28,29], our experience sheds light on two specific benefits of such approaches for mHealth apps. First, co-design activities similar to ours can help user experience designers to become familiar with medical procedures and terminology without spending too much time on secondary research. For example, in our case, the user input gathered through co-design activities remarkably eliminated the need for designers to understand the content of the laboratory service manual. Second, such activities can help designers thoroughly understand the existing user behaviors related to information usage and align the final information architecture of the mHealth app to be consistent with them. In our case, insights gained from co-design activities ultimately allowed us to design an information architecture that resonated with the one that users were accustomed to, which in turn significantly lowered the barriers for adopting the new mobile app into their daily routines.

### Role of the Facilitator in the Design Process: Facilitating Communication and Collaboration Among Multiple Stakeholders

Due to highly domain-specific content and medical terminology related to the laboratory tests, the developer team and HCI consultant had to go through a steep learning curve to familiarize themselves with the context of the mHealth app. The hierarchy, organizational structure, and bureaucracy of the hospital environment also made this process somewhat challenging for these external stakeholders. To this end, having a medical professional with experience in participatory and qualitative research as a facilitator significantly helped the external parties to overcome those challenges and understand the context quickly. Provision of an overview of the organizational structure, culture, and existing workflows, coordinating with internal stakeholders, co-conducting activities, and working around cultural barriers are some examples of the roles of the facilitator.

### Conclusions

We highly recommend a user-centered design process, based on a participatory design approach to designing mHealth apps for health professionals. Focus group discussions will be an added advantage in the design process, provided opportunity is materialized. A broad understanding of the culture, hierarchy, and bureaucracy is pivotal in planning participatory design activities; however, working on team dynamics through ice breaking and building rapport is a cornerstone of success.

In addition, a test run for polishing and fine-tuning the process is certainly a strength. Furthermore, the role of a facilitator with a medical and qualitative research background could most likely add value as well as convenience in situations where there is a steep learning curve and a race against time.

## Abbreviations

HCI: human-computer interaction
mHealth: mobile health
